# Supplementary material for: Suicide and self-harm by burns in Pakistan: a scoping review protocol
Source: BMJ Open. 2024 Mar 28;14(3):e080815. doi: 10.1136/bmjopen-2023-080815 (PMC10982739; doi:10.1136/bmjopen-2023-080815)
Supplement: Supplementary data [file bmjopen-2023-080815supp002.pdf]

Supplementary file 2 – SEARCH STRATEGY

MeSH keywords

Burns

Burns, Chemical
Burns, Electric
Burns, Inhalation

Suicide

Suicide, Attempted
Suicide, Completed

Suicide, Attempted

- Attempted Suicide
- Suicide Attempt
- Attempt, Suicide
- Parasuicide
- Parasuicides
- Self-Injurious Behavior

Suicide, Completed

- Completed Suicides
- Suicide, Fatal
- Completed Suicide
- Fatal Attempt
- Fatal Attempts
- Fatal Suicide
- Fatal Suicides
- Self-Injurious Behavior

Self-harm

- Behavior, Self-Injurious
- Self Injurious Behavior
- Self-Injurious Behaviors
- Intentional Self Injury
- Intentional Self Injuries

Noorullah A, *et al. BMJ Open* 2024; 14:e080815. doi: 10.1136/bmjopen-2023-080815

- Self Injury, Intentional
- Intentional Self Harm
- Self Harm, Intentional
- Deliberate Self-Harm
- Deliberate Self Harm
- Self-Harm, Deliberate
- Self-Injury
- Self Injury
- Self Harm
- Harm, Self
- Self-Destructive Behavior
- Behavior, Self-Destructive
- Self Destructive Behavior
- Self-Destructive Behaviors

Intentional

- Self-Injurious Behavior

Using Boolean operators:

(Self burning) OR (Self-immolation) OR (Burns) OR (self-mutilation) AND (self-harm) OR (Intentional) OR (Deliberate Self-harm) AND (suicide) OR (Attempted Suicide) OR (parasuicide) AND (Pakistan).

Search Strategy

Pubmed database

(Self burnin*) OR (Self immolation) OR (Burn*) OR (self mutilation) AND (self harm) OR (Intentio*) OR (Deliberate Self Harm) AND (suicide) OR (Attempt* Suicide) OR (Attempted Suicide) OR (parasuicide) AND (Pakistan)

Cochrane database

("Self burnin*") OR ("Self immolation") OR ("Burn*") OR ("self mutilation") AND ("self harm") OR ("Intentio*") OR ("Deliberate Self Harm") AND ("suicide*") OR ("Attempt* Suicide") OR ("parasuicide") AND (Pakistan)

Google Scholars

(“Self burnin*”) OR (“Self immolation”) OR (“Burn*”) OR (“self mutilation”) AND (“self harm”) OR (“Intentio*”) OR (“Deliberate Self Harm”) AND (“suicide*”) OR (“Attempt* Suicide”) OR (“parasuicide”) AND (Pakistan)

Pakmedinet

Suicide OR deliberate self-harm OR parasuicide OR self-harm OR attempted suicide AND burns OR self-mutilation OR Self-immolation AND Pakistan

Suicide | deliberate self-harm | parasuicide | self-harm | attempted suicide AND burns | self-mutilation | Self-immolation AND Pakistan

Grey literature for thesis and dissertation

OATD: Open Access Thesis and Dissertations.

Suicide, deliberate self-harm, self-harm, attempted suicide, burns, self-burning, Pakistan

Noorullah A, *et al. BMJ Open* 2024; 14:e080815. doi: 10.1136/bmjopen-2023-080815
