# Supplementary material for: Suicide and self-harm by burns in Pakistan: a scoping review protocol
Source: BMJ Open. 2024 Mar 28;14(3):e080815. doi: 10.1136/bmjopen-2023-080815 (PMC10982739; doi:10.1136/bmjopen-2023-080815)
Supplement: Author's manuscript [file bmjopen-2023-080815.draft_revisions.pdf]

BMJ Open

BMJ Open is committed to open peer review. As part of this commitment we make the peer review history of every article we publish publicly available.

When an article is published we post the peer reviewers' comments and the authors' responses online. We also post the versions of the paper that were used during peer review. These are the versions that the peer review comments apply to.

The versions of the paper that follow are the versions that were submitted during the peer review process. They are not the versions of record or the final published versions. They should not be cited or distributed as the published version of this manuscript.

BMJ Open is an open access journal and the full, final, typeset and author-corrected version of record of the manuscript is available on our site with no access controls, subscription charges or pay-per-view fees (http://bmjopen.bmj.com).

If you have any questions on BMJ Open's open peer review process please email info.bmjopen@bmj.com

BMJ Open

Suicide and Self-Harm by Burns in Pakistan: A Scoping Review Protocol

Journal:	*BMJ Open*
Manuscript ID	bmjopen-2023-080815
Article Type:	Protocol
Date Submitted by the Author:	11-Oct-2023
Complete List of Authors:	Noorullah, Aisha; The Aga Khan University,<br>Pirani, Shahina; The Aga Khan University, Psychiatry; Aga Khan University Hospital<br>Bebbington, Emily; Bangor University, Centre for Mental Health and Society; Ysbyty Gwynedd, Emergency Department<br>Khan, Murad ; Aga Khan University, Psychiatry
Keywords:	Suicide & self-harm < PSYCHIATRY, PUBLIC HEALTH, PSYCHIATRY

SCHOLARONE™
Manuscripts

Suicide and Self-Harm by Burns in Pakistan: A Scoping Review Protocol

Principal Investigator and Corresponding author

Dr. Aisha Noorullah
Senior Instructor
Department of Psychiatry
Aga Khan University, Stadium Road
PO Box 3500, Karachi-74800, Pakistan
Contact no. 9221-34930051
aisha.noorullah@aku.edu

Co. Authors

Shahina Pirani
Research Specialist
Department of Psychiatry
Aga Khan University Pakistan
shahina.pirani@aku.edu

Dr. Emily Bebbington
Welsh Clinical Academic Track (Public Health)
Specialist Registrar. Centre for Mental Health and Society,
Bangor University, Wrexham Academic Unit, Wrexham, LL13 7YP, UK.
e.bebbington@bangor.ac.uk

Dr. Murad M Khan
Professor Emeritus, Dept. of Psychiatry and Brain & Mind Institute
Aga Khan University Pakistan
murad.khan@aku.edu

For peer review only - http://bmjopen.bmj.com/site/about/guidelines.xhtml

ABSTRACT

Introduction

Suicide is a global public health problem. Self-inflicted burns are one of the most severe methods of suicide with high morbidity and mortality. Low and middle-income countries (LMICs) contribute 40% of all suicidal burns. Pakistan lacks comprehensive burns surveillance data that prevents the understanding of the magnitude of the problem. The aim of this scoping review is to understand the scope of the problem of suicide and self-harm using burns in Pakistan and to identify knowledge gaps within the existing literature related to this specific phenomenon.

Methods and analysis

This scoping review will follow the methodological framework proposed by Arksey and O'Malley. We will search electronic databases (PubMed, SCOPUS, Google Scholar, Pakmedinet), grey literature, and a reference list of relevant articles to identify studies for inclusion. We will look for studies on self-inflicted burns as a method of suicide and self-harm in Pakistan, published from the beginning till December 2022, in the English language. Two independent reviewers will screen all abstracts and full-text studies for inclusion. The data will be collected on a data extraction form developed through an iterative process by the research team and it will be analyzed using descriptive analysis.

Ethics and dissemination

Ethical exemption for this study has been obtained from the Institutional Review Board Committee of Aga Khan University Karachi Pakistan. The findings of the study will be disseminated by conducting workshops for stakeholders including Psychiatrists, Psychologists, counselors, general and public health physicians, and policymakers. Findings will be published in national and international peer-reviewed journals.

Key words: suicide, self-harm, burns, scoping review, Pakistan

For peer review only - http://bmjopen.bmj.com/site/about/guidelines.xhtml

Strengths and Limitations of this study

- This will be the first scoping review on suicidal burns from Pakistan.
- The review will be conducted in close collaboration between all authors. Any disagreement in the inclusion of studies will be discussed among the team until a consensus is reached. This process increases the validity and reliability of the review.
- Academic librarians will be collaborating to develop a search strategy and look for relevant papers in different databases.
- Some studies may lack essential data, making it challenging to draw comprehensive conclusions.

peer review only

For peer review only - http://bmjopen.bmj.com/site/about/guidelines.xhtml

BACKGROUND

Burns are a global public health problem, causing an estimated 180,000 deaths per year. World Health Organization (WHO) states that mortality is higher for low-middle-income countries, almost two-thirds of deaths occur in the WHO African and South-East Asia regions.[1]

Burns are catastrophic traumas, as they require prolonged hospitalization and result in physical and psychological sequelae.[2] Non-fatal burns contribute to morbidity, such as disfigurement, and disability, leading to stigma and abandonment for the survivors. Burns are among the leading causes of disability-adjusted life-years (DALYs) lost in Asia.[3]

A burn is mostly caused by heat or electricity, friction, or contact with chemicals resulting in injury to the skin or other organic tissue.[4]

Patients presenting with burns may have injuries resulting from accidents, suicide attempts, or acts of homicide. Irrespective of the mode of the burns, serious injuries requiring hospital admission remain a large problem in Pakistan because few specialist facilities are available, and the overall outcome of these patients remains poor as compared with developed countries.[5]

Suicide is a serious public health problem. Every year suicide contributes to 700,000 deaths worldwide and 77% of suicides occurred in low- and middle-income countries in 2019.[6] Self-harm is the most important risk factor for suicide in subsequent years. For each suicide, there are more than 20 suicide attempts.[7] According to the WHO Global Health Estimates,[6] 19331 [males:14,771; females:4560] suicides were reported in Pakistan, making a suicide rate of 8.9 per 100,000 population in 2019. By this estimate, 200,000-400,000 acts of self-harm occur yearly in Pakistan.

Suicide by burning is a rare condition in developed countries (0.06-1% of all suicides) but is more frequent in developing countries (accounting for as many as 40.3% of all suicides).[8] Self-burn is among the highly fatal methods of suicide with a unique geographical spread that affects less developed countries such as India, Iran, Afghanistan, and Sri Lanka.[9]

The burden of burns resulting from suicide and self-harm is substantial in South Asia. In Pakistan, a grasp of the full extent of this problem is hindered by the absence of comprehensive burns surveillance data. The lack of a national surveillance system and potential misclassification due to medicolegal processes contribute to underreporting. Overall, there is limited research available on suicide and self-harm in South Asia, with a particular focus on burns as a prevalent method. This challenge underscores the need for a deeper understanding of the issue in Pakistan.

For peer review only - http://bmjopen.bmj.com/site/about/guidelines.xhtml

Pakistan is a multiethnic country in South Asia. It is the world's fifth-most populous country, home to approximately 220 million people.[10] The predominant religion of the country is Islam, the second-largest Muslim majority**.** Pakistan has four provinces: Punjab, Sindh, Baluchistan, and the Khyber Pakhtunkhwa (KPK) plus the Islamabad Capital Territory and other administrative areas (Gilgit-Baltistan and Azad Jammu and Kashmir—AJK).[11]Around 63% of the total population is rural while 37% is found to be residing in urban areas. The population age structure is 0-14 (38%), 15-64 (58%), and 65 years and over (4%). The life expectancy at birth for women in Pakistan is approximately 65.5 years, while for men is 64.5 years. The literacy rate is around 61%.[10] As per the latest Household Integrated Economic Survey (HIES), 21.9% of the population is living below the poverty line.[12]

Pakistan is battling with diverse health challenges and has one of the poorest mental health indicators. Mental health in Pakistan is complicated by a plethora of economic, sociocultural, and religious factors, further deteriorated by the inadequacy of mental health infrastructure.[13] Of the overall health budget, the government invests 0.4% in mental health. Moreover, the shortage of qualified psychiatrists, with less than 500 available in the country, poses a significant barrier to accessible mental health care.[14]

The nation's experience of long-standing political disputes, violence, terrorism, epidemics, droughts, earthquakes, and issues related to safety and security has profound impacts in various domains.[15] These challenges have resulted in economic problems, increased poverty rates, unemployment, and notable economic disparities. All these factors collectively contribute to the emergence of psychological issues in the population and the risk of suicide and self-harm in society.

Significance of the study

The importance of reviewing the existing literature on burns studies in Pakistan lies in investigating the true extent of the burden imposed by suicidal burn injuries. It is crucial to explore the frequency, demographics, and outcomes reported to gain insights into this issue. Furthermore, the comparability of the findings in the literature remains undetermined, emphasizing the need for a scoping review to assess the availability and comparability of the existing research. Identifying specific groups that are prominently affected by these injuries may also highlight the necessity for further focused studies to address their special needs. The gravity of burns injuries, in terms of fatality,[16] and the availability of the method with their profound impact on physical and mental

For peer review only - http://bmjopen.bmj.com/site/about/guidelines.xhtml

health, differentiate these cases from other forms of self-harm, necessitates a comprehensive understanding of the under-researched subject matter in Pakistan. This scoping review will also serve as an initial step as part of the planning to commence a surveillance system in Pakistan by gauging the magnitude of the problem and its implications. A search was conducted at Open Science Framework (OSF), Figshare, PubMed, and Prospero for systematic and scoping review on self-inflicted burn injuries in Pakistan, but no prior research specific to Pakistan was identified. Existing literature reveals a scoping review[17] and a systematic review[18] on burns injuries from South Asia including Pakistan; however, the objective and time frame of those reviews varies from the current review.

Aims & Objectives

The aim of this scoping review is to understand the scope of the problem of suicide and self-harm using burns as a method of attempt across various age groups in Pakistan. It will also provide evidence to identify the knowledge gap in the existing literature on the phenomenon of interest. The specific objectives of the review include:

1. To assess the availability of data on the frequency of self-harm and suicide cases involving burns across various demographic factors in Pakistan.
2. To examine the methods commonly used for self-inflicted burns including self-harm and suicide in Pakistan.
3. To evaluate the outcomes of self-inflicted burns in Pakistan.

METHODOLOGY

To synthesize evidence on self-inflicted burns as a method of self-harm or suicide in Pakistan, a scoping review will be carried out. This scoping review will follow the methodological framework proposed by Arksey and O'Malley[19] and amended by Levac et al.[20] This framework consists of six stages: (1) Identifying the Research Question, (2) Identifying Relevant Studies, (3) Study Selection, (4) Data Charting, (5) Data Analysis and Reporting the Results, (6) Consultation The scoping review method is described considering the above six stages.

For peer review only - http://bmjopen.bmj.com/site/about/guidelines.xhtml

Stage 1: Identifying the Research Question

We identified the following research questions based on the literature review:

Research question 1A

What is the frequency of self-harm and suicide cases involving burns in Pakistan?

Research question 1B

How is the frequency of self-harm and suicide cases involving burns distributed across different demographic factors such as age, sex, and marital status in Pakistan?

Research question 2A

What are common methods used for self-inflicted burns, including self-harm and suicide, in Pakistan?

Research question 2B

Are there any variations in the methods of self-inflicted burns based on demographic factors such as age, sex, and marital status in Pakistan?

Research question 3A

What are the short-term and long-term outcomes of self-inflicted burns in Pakistan?

Research question 3B

Are there any variations in the outcomes of self-inflicted burns based on demographic factors such as age, sex, and marital status in Pakistan?

Stage 2: Identifying Relevant Studies

A robust search strategy will be developed with the help of an academic librarian and relevant studies will be identified. MESH keywords will be identified for each concept included in the review question (self-burning, suicidal, intentional) and synonyms of key terms will be used. We will use Boolean operators with a combination of terms including (Self burning) OR (Self-immolation) OR (Burns) OR (self-mutilation) AND (self-harm) OR (Intentional) OR (Deliberate Self-harm) AND (suicide) OR (Attempted Suicide) OR (parasuicide) AND (Pakistan).

We will search in four databases including the National Library of Medicine's MEDLINE (PubMed), Google scholars, Cochrane Library, and Pakmedinet. We will also search 'grey literature' that includes unpublished theses, conference papers, and other reports. In addition, an ancestry approach will be taken in which the reference lists of retrieved articles will be checked for relevant references. Databases will be searched from the beginning till December 2022,

For peer review only - http://bmjopen.bmj.com/site/about/guidelines.xhtml

For peer review only

published in the English language. The search strategies for each database will be similar in structure with similar search terms and synonyms. Additional keywords and sources identified during the process will be noted and reported. A separate record of the search results will be kept in a spreadsheet file including the date of search, search terms, and the number of retrieved articles. Search results will be exported into the reference management software Endnote and duplicate articles will be removed.

Eligibility Criteria

The selection of studies in the review will be based on thematic framework 'Population–Concept–Context (PCC)' recommended by Joanna Briggs Institute for scoping reviews,[21] outlined under broader eligibility criteria. Additional filters for type of publication and language preference would be considered (refer table 1)

Table 1: Inclusion and Exclusion Criteria for the Scoping Review

Themes	Inclusion criteria	Exclusion criteria
Population	Studies included populations with suicidal burns, in all genders, and across all ages.	Population with unintentional burns injuries.
Concept	-Studies that explicitly mentioned self-harm or completed suicide by burns as a method of choice.<br><br>-Studies that used the term intentional burns, suicidal or self-harm burns.<br><br>-Articles using different terminologies for suicidal burns (e.g. self-mutilation, self-immolation, self-inflicted burns)	Any article on burns that has no connection with suicide or self-harm.
Context	-Studies conducted within the geographical boundaries of Pakistan.<br><br>-Studies conducted at hospital or community setting.	Studies on Pakistanis residing outside the country.

For peer review only - http://bmjopen.bmj.com/site/about/guidelines.xhtml

	-Cases may be based on autopsy reports/forensic reports or Medico-legal reports.	
**Additional Filters**		
Article Type	-Original research article with any study design quantitative or qualitative study.<br><br>-Case studies, Case series or Case report<br><br>- Editorial and Short communication<br><br>- Systematic, scoping, or rapid review.<br><br>-'grey literature' that includes unpublished theses, conference papers, and other reports.	Media news
Reporting/ Language	-Articles published in the English language.	Other languages
Date of publication	Studies published from the beginning till December 2022, will be considered for this review.	Studies published in 2023

For peer review only

For peer review only - http://bmjopen.bmj.com/site/about/guidelines.xhtml

Stage 3: Study Selection

Before initiating the search, an exercise will be conducted among two researchers to ensure reliability in selecting articles for inclusion. It consists of an independent screening of a random sample of 5% of the citations by the two independent reviewers. Upon receiving low agreement among two researchers, selection criteria will be revisited.

The study selection process will take place in two steps. In the first step, the study titles and abstracts will be screened by two reviewers (SP and AN) to determine eligibility for full-text screening based on a priori inclusion criteria. A record of the search results will be kept in a spreadsheet file including date of search, search terms, and number of retrieved articles. Search results will be exported into reference management software Endnote and duplicate articles will be removed. In the second stage of the selection process, the full text of the articles will be retrieved and uploaded in an endnote, that will also be independently screened by the two reviewers (SP and AN). Any disagreements between reviewers regarding eligibility will be resolved through discussion and involving a third researcher (MK).

An adapted version of the PRISMA flow diagram will be used to report the final numbers of included studies. Reasons for excluding the studies will be recorded at the full-text screening stage.

Stage 4: Charting the Data

The data charting form has been developed in Microsoft excel sheet after consultation with the subject expert and by reviewing the existing literature (refer supplementary file 1). This form included information on the source of the study, the place where the study was conducted, study design, time of the study, number of burns cases, number of suicidal burns, mechanism of burns, age, gender, marital status, reasons for burns, and outcome. The form will be pilot-tested, and modifications will be made accordingly.

The data collection started in October 2023.

Stage 5: Data Analysis and Reporting the Results

The data will be analyzed descriptively using data charting form. The study question included the frequency of suicidal burns across various demographics, methods commonly used for self-inflicted burns, and the outcome of injury will be the basis for analysis. The characteristics

For peer review only - http://bmjopen.bmj.com/site/about/guidelines.xhtml

of the study related to suicidal burns will be presented, using tabulations or figures/flow chart where appropriate, in order to present a synthesis of key findings according to the scoping review objectives.

Reporting of Scoping Review

The scoping review will be reported according to the Preferred Reporting Items for Systematic reviews and Meta-Analyses extension for Scoping Reviews (PRISMA-ScR) Checklist (refer supplement file 2)

Stage 6: Consultations

In this final stage, consultation with the subject expert (MK, EB) will be undertaken to validate the findings and add strength to the study. The subject expert will read the results and provide feedback, adding extra value to the scoping review.

Patient and public involvement

There has been no patient or public involvement in the development of this protocol, nor will there be in the scoping review itself.

Coordination, Monitoring & Quality control

The study will ensure quality control by adhering to the standard protocols of conducting scoping review. This includes.

- Use of five stage framework by Arksey and O'Milley for conducting literature review
- Development of a comprehensive search strategy
- Training and debriefing session for the two staff on conducting scoping review.
- Independent review by two experienced team members.
- Frequent meetings with research team to review the findings.

For peer review only - http://bmjopen.bmj.com/site/about/guidelines.xhtml

Ethical Considerations and dissemination

Ethical exemption for this study has been obtained from the Institutional Review Board Committee of Aga Khan University Karachi Pakistan. The findings of the study will be disseminated by conducting workshops for stakeholders including Psychiatrists, Psychologists, counselors, general and public health physicians, and policymakers. Findings will be published in national and international peer-reviewed journals.

For peer review only - http://bmjopen.bmj.com/site/about/guidelines.xhtml

Authors' Contributions

AN & MK conceived the study, involved in concept mapping and planning of the study. SP looked for literature review and developed a study tool. SP & AN contributed to writing the study protocol. EB critically reviewed the protocol. MK intellectually contributed to the study. All authors have read and approved the final manuscript.

Funding

This review received no specific grant from any funding agency in the public, commercial or not-for-profit sectors.

Competing interests

None declared.

Patient consent for publication

Not required.

Ethics approval

Study protocol is exempted by Aga Khan University Ethical Review Committee with ERC # 2023-9102-26269

For peer review only

For peer review only - http://bmjopen.bmj.com/site/about/guidelines.xhtml

REFERENCES

1. World Health Organization. Burns. Geneva: World Health Organization; 2018 https://www.who.int/news-room/fact-sheets/detail/burns

2. Spronk I, Legemate C, Oen I, van Loey N, Polinder S, van Baar M. Health related quality of life in adults after burn injuries: a systematic review. PloS one. 2018; 13(5):e0197507.

3. Collier ZJ, McCool K, Magee III WP, Potokar T, Gillenwater J. 58 Burn injuries in Asia: a global burden of disease study. Journal of Burn Care & Research. 2022;43(Supplement_1):S40-1.

4. Burns - Symptoms and causes. https://www.mayoclinic.org/diseases-conditions/burns/symptoms-causes/syc-20370539

5. Khan N, Malik MN. Presentation of burn injuries and their management outcome. Journal-Pakistan Medical Association. 2006;56(9):394.

6. World Health Organization. Suicide worldwide in 2019: global health estimates. Geneva: World Health Organization; 2021.

7. Owens D, Horrocks J, House A. Fatal and non-fatal repetition of self-harm: systematic review. The British Journal of Psychiatry. 2002;181(3):193-9.

8. Ahmadi A. Suicide by self-immolation: comprehensive overview, experiences and suggestions. Journal of burn care & research. 2007;28(1):30-41.

9. Mohammadi AA, Danesh N, Sabet B, Amini M, Jalaeian H. Self-inflicted burn injuries in southwest Iran. Journal of burn care & research. 2008;29(5):778-83.

10. Pakistan Bureau of Statistics. Pakistan Demographic survey 2020. Government of Pakistan, 2022. www.pbs.gov.pk

11. National Institute of Population Studies. Pakistan Demographic and Health Survey 2017-18. Islamabad, Pakistan, and Rockville, Maryland, USA: NIPS and ICF, 2019.

12. Shehzad, R. 22% Pakistanis living below poverty line. The Express Tribune. (2021, Sept 21). https://tribune.com.pk/story/2322313/22-pakistanis-living-below-poverty-line-na-told

For peer review only - http://bmjopen.bmj.com/site/about/guidelines.xhtml

13. Ali TM, Gul S. Community mental health services in Pakistan: Review study from Muslim world 2000-2015. Psychology, Community & Health. 2018;7(1).57–71.

14. Bhatti, M.W. Pakistan needs more trained Psychiatrists. International The News. (2020, March 01). https://www.thenews.com.pk/print/621912-pakistan-needs-more-trained-psychiatrists.

15. Abbas T, Jamil S. Psychological consequences of terrorism: case study of Pakistan. Margalla Papers, 2019, 23, 71-79.

16. Ali SA, Hamiz-Ul-Fawwad S, Al-Ibran E, Ahmed G, Saleem A, Mustafa D, Hussain M. Clinical and demographic features of burn injuries in karachi: a six-year experience at the burns centre, civil hospital, Karachi. Annals of burns and fire disasters. 2016;29(1):4.

17. Bebbington E, Ramesh P, Kakola M, McPhillips R, Bibi F, Hanif A, Morris N, Khan M, Poole R, Robinson C. Terminology and methods used to differentiate injury intent of hospital burn patients in South Asia: a systematic scoping review protocol. Systematic reviews. 2023;12(1):153.

18. Golshan A, Patel C, Hyder AA. A systematic review of the epidemiology of unintentional burn injuries in South Asia. Journal of public health. 2013;35(3):384-96.

19. Arksey H, O'Malley L. Scoping studies: towards a methodological framework. Int J Soc Res Methodol. 2005;8:19–3

20. Levac D, Colquhoun H, O'Brien KK. Scoping studies: advancing the methodology. Implement Sci. 2010;5:69

21. Peters MD, Marnie C, Tricco AC, Pollock D, Munn Z, Alexander L, McInerney P, Godfrey CM, Khalil H. Updated methodological guidance for the conduct of scoping reviews. JBI evidence synthesis. 2020;18(10):2119-26.

For peer review only - http://bmjopen.bmj.com/site/about/guidelines.xhtml

Studies on Self-burning from Pakistan

Author/year	Study Design	Year of Study	Source of data	Setting (City/province)

For peer review only

Suicide/SH cases	Total number of Burns cases	Number of Suicidal burns	Age

For peer review only

Gender	Marital status	Occupation	Place of burns	Extend of burns (TBSA)	Depth of burns

For peer review only

Reasons	Method/mechanism of burns	Outcome	Notes

For peer review only

Preferred Reporting Items for Systematic reviews and Meta-Analyses extension for Scoping Reviews (PRISMA-ScR) Checklist

SECTION	ITEM	PRISMA-ScR CHECKLIST ITEM	REPORTED ON PAGE #
**TITLE**			
Title	1	Identify the report as a scoping review.	
**ABSTRACT**			
Structured summary	2	Provide a structured summary that includes (as applicable): background, objectives, eligibility criteria, sources of evidence, charting methods, results, and conclusions that relate to the review questions and objectives.	
**INTRODUCTION**			
Rationale	3	Describe the rationale for the review in the context of what is already known. Explain why the review questions/objectives lend themselves to a scoping review approach.	
Objectives	4	Provide an explicit statement of the questions and objectives being addressed with reference to their key elements (e.g., population or participants, concepts, and context) or other relevant key elements used to conceptualize the review questions and/or objectives.	
**METHODS**			
Protocol and registration	5	Indicate whether a review protocol exists; state if and where it can be accessed (e.g., a Web address); and if available, provide registration information, including the registration number.	
Eligibility criteria	6	Specify characteristics of the sources of evidence used as eligibility criteria (e.g., years considered, language, and publication status), and provide a rationale.	
Information sources*	7	Describe all information sources in the search (e.g., databases with dates of coverage and contact with authors to identify additional sources), as well as the date the most recent search was executed.	
Search	8	Present the full electronic search strategy for at least 1 database, including any limits used, such that it could be repeated.	
Selection of sources of evidence†	9	State the process for selecting sources of evidence (i.e., screening and eligibility) included in the scoping review.	
Data charting process‡	10	Describe the methods of charting data from the included sources of evidence (e.g., calibrated forms or forms that have been tested by the team before their use, and whether data charting was done independently or in duplicate) and any processes for obtaining and confirming data from investigators.	
Data items	11	List and define all variables for which data were sought and any assumptions and simplifications made.	
Critical appraisal of individual sources of evidence§	12	If done, provide a rationale for conducting a critical appraisal of included sources of evidence; describe the methods used and how this information was used in any data synthesis (if appropriate).	
Synthesis of results	13	Describe the methods of handling and summarizing the data that were charted.	

For peer review only

SECTION	ITEM	PRISMA-ScR CHECKLIST ITEM	REPORTED ON PAGE #
**RESULTS**			
Selection of sources of evidence	14	Give numbers of sources of evidence screened, assessed for eligibility, and included in the review, with reasons for exclusions at each stage, ideally using a flow diagram.	
Characteristics of sources of evidence	15	For each source of evidence, present characteristics for which data were charted and provide the citations.	
Critical appraisal within sources of evidence	16	If done, present data on critical appraisal of included sources of evidence (see item 12).	
Results of individual sources of evidence	17	For each included source of evidence, present the relevant data that were charted that relate to the review questions and objectives.	
Synthesis of results	18	Summarize and/or present the charting results as they relate to the review questions and objectives.	
**DISCUSSION**			
Summary of evidence	19	Summarize the main results (including an overview of concepts, themes, and types of evidence available), link to the review questions and objectives, and consider the relevance to key groups.	
Limitations	20	Discuss the limitations of the scoping review process.	
Conclusions	21	Provide a general interpretation of the results with respect to the review questions and objectives, as well as potential implications and/or next steps.	
**FUNDING**			
Funding	22	Describe sources of funding for the included sources of evidence, as well as sources of funding for the scoping review. Describe the role of the funders of the scoping review.	

JBI = Joanna Briggs Institute; PRISMA-ScR = Preferred Reporting Items for Systematic reviews and Meta-Analyses extension for Scoping Reviews.

* Where *sources of evidence* (see second footnote) are compiled from, such as bibliographic databases, social media platforms, and Web sites.

† A more inclusive/heterogeneous term used to account for the different types of evidence or data sources (e.g., quantitative and/or qualitative research, expert opinion, and policy documents) that may be eligible in a scoping review as opposed to only studies. This is not to be confused with *information sources* (see first footnote).

‡ The frameworks by Arksey and O'Malley (6) and Levac and colleagues (7) and the JBI guidance (4, 5) refer to the process of data extraction in a scoping review as data charting.

§ The process of systematically examining research evidence to assess its validity, results, and relevance before using it to inform a decision. This term is used for items 12 and 19 instead of "risk of bias" (which is more applicable to systematic reviews of interventions) to include and acknowledge the various sources of evidence that may be used in a scoping review (e.g., quantitative and/or qualitative research, expert opinion, and policy document).

From: Tricco AC, Lillie E, Zarin W, O'Brien KK, Colquhoun H, Levac D, et al. PRISMA Extension for Scoping Reviews (PRISMAScR): Checklist and Explanation. Ann Intern Med. 2018;169:467–473. doi: 10.7326/M18-0850.

For peer review only - http://bmjopen.bmj.com/site/about/guidelines.xhtml

BMJ Open

Suicide and Self-Harm by Burns in Pakistan: A Scoping Review Protocol

Journal:	*BMJ Open*
Manuscript ID	bmjopen-2023-080815.R1
Article Type:	Protocol
Date Submitted by the Author:	20-Dec-2023
Complete List of Authors:	Noorullah, Aisha; The Aga Khan University<br>Pirani, Shahina; The Aga Khan University<br>Bebbington, Emily; Bangor University, Centre for Mental Health and Society; Ysbyty Gwynedd, Emergency Department<br>Khan, Murad ; The Aga Khan University
**Primary Subject Heading**:	Mental health
Secondary Subject Heading:	Public health
Keywords:	Suicide & self-harm < PSYCHIATRY, PUBLIC HEALTH, PSYCHIATRY

SCHOLARONE™
Manuscripts

Suicide and Self-Harm by Burns in Pakistan: A Scoping Review Protocol

Principal Investigator

Dr. Aisha Noorullah
Senior Instructor
Department of Psychiatry
Aga Khan University, Stadium Road
PO Box 3500, Karachi-74800, Pakistan
Contact no. 9221-34930051
aisha.noorullah@aku.edu

Co. Investigators

Ms. Shahina Pirani
Research Specialist
Department of Psychiatry
Aga Khan University Pakistan
shahina.pirani@aku.edu

Dr. Emily Bebbington*
Welsh Clinical Academic Track (Public Health)
Specialist Registrar. Centre for Mental Health and Society,
Bangor University, Wrexham Academic Unit, Wrexham, LL13 7YP, UK.
e.bebbington@bangor.ac.uk

Dr. Murad M Khan
Professor Emeritus, Dept. of Psychiatry and Brain & Mind Institute
Aga Khan University Pakistan
murad.khan@aku.edu

***Corresponding authors**
Dr. Emily Bebbington, e.bebbington@bangor.ac.uk

For peer review only

For peer review only - http://bmjopen.bmj.com/site/about/guidelines.xhtml

ABSTRACT

Introduction

Suicide is a global public health problem. Self-inflicted burns are one of the most severe methods of suicide with high morbidity and mortality. Low and middle-income countries (LMICs) contribute 40% of all suicidal burns. Pakistan lacks comprehensive burns surveillance data which prevents the understanding of the magnitude of the problem. This scoping review aims to understand the scope of the problem of suicide and self-harm using burns in Pakistan and to identify knowledge gaps within the existing literature related to this specific phenomenon.

Methods and analysis

This scoping review will follow the methodological framework proposed by Arksey and O'Malley. We will search electronic databases (PubMed, Cochrane, Google Scholar, Pakmedinet), grey literature, and a reference list of relevant articles to identify studies for inclusion. We will look for studies on self-inflicted burns as a method of suicide and self-harm in Pakistan, published from the beginning till December 2022, in the English language. Two independent reviewers will screen all abstracts and full-text studies for inclusion. The data will be collected on a data extraction form developed through an iterative process by the research team and it will be analyzed using descriptive analysis.

Ethics and dissemination

Ethical exemption for this study has been obtained from the Institutional Review Board Committee of Aga Khan University Karachi Pakistan. The findings of the study will be disseminated by conducting workshops for stakeholders including Psychiatrists, Psychologists, counselors, general and public health physicians, and policymakers. Findings will be published in national and international peer-reviewed journals.

Keywords: suicide, self-harm, burns, scoping review, Pakistan

For peer review only - http://bmjopen.bmj.com/site/about/guidelines.xhtml

STRENGTHS AND LIMITATIONS OF THIS STUDY

- PRISMA protocol and scoping review reporting guidelines have been followed in the preparation of this manuscript and will be followed throughout the scoping review process.
- The review will be conducted in close collaboration between all authors. Any disagreement in the inclusion of studies will be discussed among the team until a consensus is reached. This process increases the validity and reliability of the review.
- Academic librarians will be collaborating to develop a search strategy and look for relevant papers in different databases.
- Only English language studies will be included. This is the dominant language used for the practice of medicine in Pakistan, but it may restrict the breadth of our results.

peer review only

For peer review only - http://bmjopen.bmj.com/site/about/guidelines.xhtml

BACKGROUND

Burns are a global public health problem, causing an estimated 180,000 deaths per year (1). Mortality from burn injuries is higher in low and middle-income countries (LMICs). Almost two-thirds of deaths occur in the WHO African and South-East Asia regions (1).

Self-inflicted burns are one of the most painful and violent methods of suicide, with high morbidity and mortality (2). Suicide by burning is a rare condition in high-income countries (0.06-1% of all suicides) but is more frequent in LMICs (accounting for as many as 40.3% of all suicides) (3). 77% of all suicides occurred in LMICs in 2019 (4).

Burn injuries can result in prolonged hospitalization and result in long-term physical and psychological sequelae (5). Non-fatal burns contribute to morbidity, such as disfigurement, and disability, leading to stigma and abandonment for the survivors (1). Burns are among the leading causes of disability-adjusted life-years (DALYs) lost in Asia (6).

Pakistan, a South Asian Islamic country, there are no official statistics on suicides at the national level (7). The WHO estimated there were 19,331 suicidal deaths and a rate of 8.9/100,000 people in 2019 (4). Until recently, suicides were treated as criminal acts in Pakistan, a religiously condemned and highly stigmatized. In December 2022, suicide and self-harms acts were decriminalized (8). However, it will take time for the abolition of the legal processes associated with them. Suicide and self-inflicted injuries are underreported due to religious, socio-cultural, and medicolegal processes. Suicidal burn injury is an important but under-researched area in Pakistan. Due to fear of societal stigma and legal issues, families withhold information about assault and self-harm and label it as accidental. This led to misclassification and undercounting of cases (9,10).

There are few systematic reviews and meta-analyses on suicidal burns from various parts of the world (11-14). These reviews revealed that highest prevalence of suicidal burns is found to be in South Asian countries including India, Iran, Iraq, Afghanistan, and Sri Lanka. In these studies, women represent many cases of suicidal burning with high mortality. Women who engage in suicidal self-burning were typically young, married, and mothers (2,11,12,14).

Existing literature reveals a systematic scoping review protocol on burns injury from South Asia (15). This review focused on terminology and methods used to differentiate the intent of hospital presenting burn injuries in South Asia. But there is no study that we are aware of that systematically investigates the demographics and outcomes of all suicidal burns in Pakistan.

For peer review only - http://bmjopen.bmj.com/site/about/guidelines.xhtml

The importance of reviewing the existing literature on burns studies in Pakistan lies in investigating the true extent of the burden imposed by suicidal burn injuries. It is crucial to explore the frequency, demographics, and outcomes reported to gain insights into this issue. Furthermore, the comparability of the findings in the literature remains undetermined, emphasizing the need for a scoping review to assess the availability and comparability of the existing research. Identifying specific groups that are prominently affected by these injuries may also highlight the necessity for further focused studies to address their special needs. The gravity of burns injuries, in terms of fatality (16), and the availability of the method with their profound impact on physical and mental health, differentiate these cases from other forms of self-harm, necessitates a comprehensive understanding of the under-researched subject matter in Pakistan. This scoping review will also serve as an initial step as part of the planning to commence a surveillance system in Pakistan by gauging the magnitude of the problem and its implications.

A search was conducted at Open Science Framework (OSF), Figshare, PubMed, and Prospero for systematic and scoping review on self-inflicted burn injuries in Pakistan, but no prior research specific to Pakistan was identified.

Aims & Objectives

This scoping review aims to understand the scope of the problem of suicide and self-harm using burns as a method of attempt across various age groups in Pakistan. It will also provide evidence to identify the knowledge gap in the existing literature on the phenomenon of interest. The specific objectives of the review include:

1. To assess the availability of data on the frequency of self-harm and suicide cases involving burns across various demographic factors in Pakistan.
2. To examine the methods commonly used for self-inflicted burns including self-harm and suicide in Pakistan.
3. To evaluate the outcomes of self-inflicted burns in Pakistan.

METHODS

We will conduct a scoping review to synthesize evidence on self-inflicted burns as a method of self-harm or suicide in Pakistan. This protocol has been written using Preferred Reporting Items for Systematic Reviews and Meta-Analyses extension for Scoping Reviews (PRISMA-ScR) (refer

For peer review only - http://bmjopen.bmj.com/site/about/guidelines.xhtml

supplementary file 1). This scoping review will follow the methodological framework proposed by Arksey and O'Malley (17) and amended by Levac et al. (18) This framework consists of six stages: (1) Identifying the Research Question, (2) Identifying Relevant Studies, (3) Study Selection, (4) Data Charting, (5) Data Analysis and Reporting the Results, (6) Consultation

The scoping review method is described considering the above six stages. This review has been registered with the Open Science Framework: https://doi.org/10.17605/OSF.IO/XA68S.

Stage 1: Identifying the Research Question

Based on the literature review, we formulated the following research questions:

Research question 1A

What is the frequency of self-harm and suicide cases involving burns in Pakistan?

Research question 1B

How is the frequency of self-harm and suicide cases involving burns distributed across different demographic factors such as age, sex, and marital status in Pakistan?

Research question 2A

What are common methods used for self-inflicted burns, including self-harm and suicide, in Pakistan?

Research question 2B

Are there any variations in the methods of self-inflicted burns based on demographic factors such as age, sex, and marital status in Pakistan?

Research question 3A

What are the short-term and long-term outcomes of self-inflicted burns in Pakistan?

Research question 3B

Are there any variations in the outcomes of self-inflicted burns based on demographic factors such as age, sex, and marital status in Pakistan?

Stage 2: Identifying Relevant Studies

For identifying the relevant studies, an academic librarian will assist in developing a robust search strategy. We will identify MESH keywords for each concept (burns, suicide, self-harm, intentional) included in the review question and use synonyms of key terms. Additionally, we will use Boolean operators with a combination of terms including (Self burning) OR (Self-immolation)

For peer review only - http://bmjopen.bmj.com/site/about/guidelines.xhtml

OR (Burns) OR (self-mutilation) AND (self-harm) OR (Intentional) OR (Deliberate Self-harm) AND (suicide) OR (Attempted Suicide) OR (parasuicide) AND (Pakistan) to look for relevant literature (refer supplementary file 2).

We will search in four databases including the National Library of Medicine's MEDLINE (PubMed), Google Scholar, Cochrane Library, and Pakmedinet. We will also search 'grey literature' that includes unpublished theses, conference papers, and other reports. In addition, we will employ an ancestry approach, checking the reference lists of retrieved articles for relevant references. We will search databases from the beginning until December 2022, published in the English language. The search strategies for each database will be similar in structure with similar search terms and synonyms. Additional keywords and sources identified during the process will be noted and reported. We will keep a separate record of the search results in a spreadsheet file including the date of search, search terms, and the number of retrieved articles. The search results will be exported into the reference management software Endnote and duplicate articles will be removed.

Eligibility Criteria

The selection of studies in the review will be based on thematic framework 'Population–Concept–Context (PCC)' recommended by Joanna Briggs Institute for scoping reviews (19), outlined under broader eligibility criteria. Consideration would be given to additional filters for type of publication and language preference (refer table 1)

Table 1: Inclusion and Exclusion Criteria for the Scoping Review

Themes	Inclusion criteria	Exclusion criteria
Population	Studies included populations with suicidal burns, in all genders, and across all ages.	Population with unintentional burns injuries.
Concept	-Studies that explicitly focused on burns as a method of self-harm or suicide.<br><br>-Studies focused on self-harm and suicide where burns are used as one of the methods.	Any article on burns that has no connection with suicide or self-harm.

For peer review only - http://bmjopen.bmj.com/site/about/guidelines.xhtml

	-Studies that used the term intentional burns, suicidal or self-harm burns.<br><br>-Articles using different terminologies for suicidal burns (e.g. self-mutilation, self-immolation, self-inflicted burns)	
Context	-Studies conducted within the geographical boundaries of Pakistan.<br><br>-Studies conducted at hospital or community setting.<br><br>-Cases may be based on autopsy reports/forensic reports or Medico-legal reports.	Studies on Pakistanis residing outside the country.
**Additional Filters**		
Article Type	-Original research article with any study design quantitative or qualitative study.<br><br>-Case studies, Case series or Case report<br><br>- Editorial and Short communication<br><br>- Systematic, scoping, or rapid review.<br><br>-'grey literature' that includes unpublished theses, conference papers, and other reports.	Media news
Reporting/ Language	-Articles published in the English language.	Other languages
Date of publication	Studies published from the beginning till December 2022, will be considered for this review.	Studies published after December 2022

Stage 3: Study Selection

Two researchers will conduct an exercise before initiating the search to ensure reliability in selecting articles for inclusion. This exercise will involve the independent screening of a random

For peer review only - http://bmjopen.bmj.com/site/about/guidelines.xhtml

sample of 5% of the citations by the two independent reviewers. If the two researchers show low agreement, they will revisit the selection criteria.

The study selection process involves two steps. In the first step, two reviewers (SP and AN) will screen for the study titles and abstracts to determine eligibility for full-text screening based on predefined inclusion criteria. A spreadsheet file will be used to record the search results, including the search date, terms used, and the number of retrieved articles. After exporting the search results into the Endnote reference management software, duplicate articles will be removed. In the second stage of the selection process, the full text of the articles will be retrieved and uploaded in an endnote. These will undergo independent screening by the same two reviewers (SP and AN) and any disagreements in eligibility between the reviewers will be resolved through discussion and involving a third researcher (MK).

We will use an adapted version of the PRISMA flow diagram to report the final numbers of included studies. At the full-text screening stage, we will record the reasons for excluding the studies.

Stage 4: Charting the Data

The data charting form has been developed in Microsoft Excel sheet after consultation with the subject expert and by reviewing the existing literature (refer supplementary file 3). This form included information on the source of the study, the place where the study was conducted, study design, time of the study, number of burns cases, number of suicidal burns, mechanism of burns, age, gender, marital status, reasons for burns, and outcome. We will pre-test the form and modify it accordingly.

Stage 5: Data Analysis and Reporting the Results

We will analyze the data descriptively using a data charting form. The study question included the frequency of suicidal burns across various demographics, methods commonly used for self-inflicted burns, and the outcome of injury will be the basis for analysis. We will present the characteristics of the study related to suicidal burns using tabulations or figures/flow chart where appropriate, to present a synthesis of key findings according to the scoping review objectives.

For peer review only - http://bmjopen.bmj.com/site/about/guidelines.xhtml

The researchers will report the scoping review according to the Preferred Reporting Items for Systematic Reviews and Meta-Analyses extension for Scoping Reviews (PRISMA-ScR) Checklist.

Stage 6: Consultations

In this final stage, we will consult with the subject experts (MK, EB) to validate the findings and add strength to the study. The subject expert will read the results and provide feedback, adding extra value to the scoping review.

Our scoping review work is in the study selection phase that began in November 2023. The authors anticipate that this review will be completed and submitted for publication by March 2024.

Patient and public involvement

There has been no patient or public involvement in the development of this protocol, nor will there be in the scoping review itself.

Coordination, Monitoring & Quality control

The study will ensure quality control by adhering to the standard protocols of conducting scoping reviews. This includes.

- Use of five stage framework by Arksey and O'Malley for conducting a literature review
- Development of a comprehensive search strategy
- Training and debriefing session for the two staff on conducting the scoping review.
- Independent review by two experienced team members.
- Frequent meetings with the research team to review the findings.

Ethical Considerations and Dissemination

Ethical exemption for this study has been obtained from the Institutional Review Board Committee of Aga Khan University Karachi Pakistan. We will disseminate the findings of the study by conducting workshops for stakeholders including Psychiatrists, Psychologists,

For peer review only - http://bmjopen.bmj.com/site/about/guidelines.xhtml

counselors, general and public health physicians, and policymakers. Additionally, we will publish the findings in national and international peer-reviewed journals.

For peer review only

For peer review only - http://bmjopen.bmj.com/site/about/guidelines.xhtml

Authors' Contributions

AN & MK conceived the study and were involved in concept mapping and planning of the study. SP looked for a literature review and developed a study tool. SP & AN contributed to writing the study protocol. EB critically reviewed the protocol. MK intellectually contributed to the study. All authors have read and approved the final manuscript.

Funding

This work was supported by the South Asia Self-Harm Initiative. The South Asia Self-Harm initiative is funded by the Global Challenges Research Fund, UK Research and Innovation. Project number: MR/P028144/2. Principal Investigator: Prof. Catherine Robinson. The funder has not been involved in the development of this protocol.

Competing interests

None declared.

Patient consent for publication

Not required.

Ethics approval

Study protocol is exempted by the Aga Khan University Ethical Review Committee with ERC # 2023-9102-26269

For peer review only

For peer review only - http://bmjopen.bmj.com/site/about/guidelines.xhtml

REFERENCES

1. World Health Organization. Burns. Geneva: World Health Organization; 2023 https://www.who.int/news-room/fact-sheets/detail/burns

2. Canetto SS, Pouradeli S, Khan MM, Rezaeian M. Suicidal self-burning in women and men around the world: a cultural and gender analysis of patterns and explanations. InSuicide Risk Assessment and Prevention 2022 Sep 28 (pp. 1-28). Cham: Springer International Publishing.

3. Ahmadi A. Suicide by self-immolation: comprehensive overview, experiences and suggestions. Journal of burn care & research. 2007;28(1):30-41.

4. World Health Organization. Suicide worldwide in 2019: global health estimates. Geneva: World Health Organization; 2021.

5. Spronk I, Legemate C, Oen I, van Loey N, Polinder S, van Baar M. Health related quality of life in adults after burn injuries: a systematic review. PloS one. 2018; 13(5):e0197507.

6. Collier ZJ, McCool K, Magee III WP, Potokar T, Gillenwater J. 58 Burn injuries in Asia: a global burden of disease study. Journal of Burn Care & Research. 2022;43(Supplement_1):S40-1.

7. Asad N, Pirani S, Tariq S, et al. Patterns of suicide and self-harm in Pakistan: a retrospective descriptive study protocol. BMJ Open 2022;12:e064535. doi:10.1136/ bmjopen-2022-064535

8. The Express Tribune. Law penalising suicide attempts abolished; December 23, 2022. https://tribune.com.pk/story/2392477/law-penalising-suicide-attempts-abolished

9. Naveed S, Tahir SM, Imran N, Rafiq B, Ayub M, Haider II, Khan MM. Sociodemographic characteristics and patterns of suicide in Pakistan: an analysis of current trends. Community mental health journal. 2023 Aug;59(6):1064-70.

10. Shahid M, Hyder AA. Deliberate self-harm and suicide: a review from Pakistan. International journal of injury control and safety promotion. 2008 Dec 1;15(4):233-41.

11. Laloë V. Patterns of deliberate self-burning in various parts of the world: a review. Burns. 2004;30:207–15.

For peer review only - http://bmjopen.bmj.com/site/about/guidelines.xhtml

12. Poeschla B, Combs H, Livingstone S, Romm S, Klein MB. Self-immolation: socioeconomic, cultural and psychiatric patterns. Burns. 2011;37:1049–57.

13. Hahn AP, Jochai D, Caufield-Noll CP, Hunt CA, Allen LE, Rios R, Cordts GA. Self-inflicted burns: a systematic review of the literature. J Burn Care Res. 2014;35:102–119.

14. Saadati M, Azami-Aghdash S, Heydari M, Derakhshani N, Rezapour R. Self-immolation in Iran: systematic review and meta-analysis. Bull Emerg Trauma. 2019;7:1–8.

15. Bebbington E, Ramesh P, Kakola M, McPhillips R, Bibi F, Hanif A, Morris N, Khan M, Poole R, Robinson C. Terminology and methods used to differentiate injury intent of hospital burn patients in South Asia: a systematic scoping review protocol. Systematic reviews. 2023;12(1):153.

16. Ali SA, Hamiz-Ul-Fawwad S, Al-Ibran E, Ahmed G, Saleem A, Mustafa D, Hussain M. Clinical and demographic features of burn injuries in karachi: a six-year experience at the burns centre, civil hospital, Karachi. Annals of burns and fire disasters. 2016;29(1):4.

17. Arksey H, O'Malley L. Scoping studies: towards a methodological framework. Int J Soc Res Methodol. 2005;8:19–3

18. Levac D, Colquhoun H, O'Brien KK. Scoping studies: advancing the methodology. Implement Sci. 2010;5:69

19. Peters MD, Marnie C, Tricco AC, Pollock D, Munn Z, Alexander L, McInerney P, Godfrey CM, Khalil H. Updated methodological guidance for the conduct of scoping reviews. JBI evidence synthesis. 2020;18(10):2119-26.

For peer review only

Preferred Reporting Items for Systematic reviews and Meta-Analyses extension for Scoping Reviews (PRISMA-ScR) Checklist

SECTION	ITEM	PRISMA-ScR CHECKLIST ITEM	REPORTED ON PAGE #
**TITLE**			
Title	1	Identify the report as a scoping review.	
**ABSTRACT**			
Structured summary	2	Provide a structured summary that includes (as applicable): background, objectives, eligibility criteria, sources of evidence, charting methods, results, and conclusions that relate to the review questions and objectives.	
**INTRODUCTION**			
Rationale	3	Describe the rationale for the review in the context of what is already known. Explain why the review questions/objectives lend themselves to a scoping review approach.	
Objectives	4	Provide an explicit statement of the questions and objectives being addressed with reference to their key elements (e.g., population or participants, concepts, and context) or other relevant key elements used to conceptualize the review questions and/or objectives.	
**METHODS**			
Protocol and registration	5	Indicate whether a review protocol exists; state if and where it can be accessed (e.g., a Web address); and if available, provide registration information, including the registration number.	
Eligibility criteria	6	Specify characteristics of the sources of evidence used as eligibility criteria (e.g., years considered, language, and publication status), and provide a rationale.	
Information sources*	7	Describe all information sources in the search (e.g., databases with dates of coverage and contact with authors to identify additional sources), as well as the date the most recent search was executed.	
Search	8	Present the full electronic search strategy for at least 1 database, including any limits used, such that it could be repeated.	
Selection of sources of evidence†	9	State the process for selecting sources of evidence (i.e., screening and eligibility) included in the scoping review.	
Data charting process‡	10	Describe the methods of charting data from the included sources of evidence (e.g., calibrated forms or forms that have been tested by the team before their use, and whether data charting was done independently or in duplicate) and any processes for obtaining and confirming data from investigators.	
Data items	11	List and define all variables for which data were sought and any assumptions and simplifications made.	
Critical appraisal of individual sources of evidence§	12	If done, provide a rationale for conducting a critical appraisal of included sources of evidence; describe the methods used and how this information was used in any data synthesis (if appropriate).	
Synthesis of results	13	Describe the methods of handling and summarizing the data that were charted.	

For peer review only

SECTION	ITEM	PRISMA-ScR CHECKLIST ITEM	REPORTED ON PAGE #
**RESULTS**			
Selection of sources of evidence	14	Give numbers of sources of evidence screened, assessed for eligibility, and included in the review, with reasons for exclusions at each stage, ideally using a flow diagram.	
Characteristics of sources of evidence	15	For each source of evidence, present characteristics for which data were charted and provide the citations.	
Critical appraisal within sources of evidence	16	If done, present data on critical appraisal of included sources of evidence (see item 12).	
Results of individual sources of evidence	17	For each included source of evidence, present the relevant data that were charted that relate to the review questions and objectives.	
Synthesis of results	18	Summarize and/or present the charting results as they relate to the review questions and objectives.	
**DISCUSSION**			
Summary of evidence	19	Summarize the main results (including an overview of concepts, themes, and types of evidence available), link to the review questions and objectives, and consider the relevance to key groups.	
Limitations	20	Discuss the limitations of the scoping review process.	
Conclusions	21	Provide a general interpretation of the results with respect to the review questions and objectives, as well as potential implications and/or next steps.	
**FUNDING**			
Funding	22	Describe sources of funding for the included sources of evidence, as well as sources of funding for the scoping review. Describe the role of the funders of the scoping review.	

JBI = Joanna Briggs Institute; PRISMA-ScR = Preferred Reporting Items for Systematic reviews and Meta-Analyses extension for Scoping Reviews.

* Where *sources of evidence* (see second footnote) are compiled from, such as bibliographic databases, social media platforms, and Web sites.

† A more inclusive/heterogeneous term used to account for the different types of evidence or data sources (e.g., quantitative and/or qualitative research, expert opinion, and policy documents) that may be eligible in a scoping review as opposed to only studies. This is not to be confused with *information sources* (see first footnote).

‡ The frameworks by Arksey and O'Malley (6) and Levac and colleagues (7) and the JBI guidance (4, 5) refer to the process of data extraction in a scoping review as data charting.

§ The process of systematically examining research evidence to assess its validity, results, and relevance before using it to inform a decision. This term is used for items 12 and 19 instead of "risk of bias" (which is more applicable to systematic reviews of interventions) to include and acknowledge the various sources of evidence that may be used in a scoping review (e.g., quantitative and/or qualitative research, expert opinion, and policy document).

From: Tricco AC, Lillie E, Zarin W, O'Brien KK, Colquhoun H, Levac D, et al. PRISMA Extension for Scoping Reviews (PRISMAScR): Checklist and Explanation. Ann Intern Med. 2018;169:467–473. doi: 10.7326/M18-0850.

For peer review only - http://bmjopen.bmj.com/site/about/guidelines.xhtml

Supplementary file 2 – SEARCH STRATEGY

MeSH keywords

Burns

Burns, Chemical
Burns, Electric
Burns, Inhalation

Suicide

Suicide, Attempted
Suicide, Completed

Suicide, Attempted

- Attempted Suicide
- Suicide Attempt
- Attempt, Suicide
- Parasuicide
- Parasuicides
- Self-Injurious Behavior

Suicide, Completed

- Completed Suicides
- Suicide, Fatal
- Completed Suicide
- Fatal Attempt
- Fatal Attempts
- Fatal Suicide
- Fatal Suicides
- Self-Injurious Behavior

Self-harm

- Behavior, Self-Injurious
- Self Injurious Behavior
- Self-Injurious Behaviors
- Intentional Self Injury
- Intentional Self Injuries

For peer review only - http://bmjopen.bmj.com/site/about/guidelines.xhtml

- Self Injury, Intentional
- Intentional Self Harm
- Self Harm, Intentional
- Deliberate Self-Harm
- Deliberate Self Harm
- Self-Harm, Deliberate
- Self-Injury
- Self Injury
- Self Harm
- Harm, Self
- Self-Destructive Behavior
- Behavior, Self-Destructive
- Self Destructive Behavior
- Self-Destructive Behaviors

Intentional

- Self-Injurious Behavior

Using Boolean operators:

(Self burning) OR (Self-immolation) OR (Burns) OR (self-mutilation) AND (self-harm) OR (Intentional) OR (Deliberate Self-harm) AND (suicide) OR (Attempted Suicide) OR (parasuicide) AND (Pakistan).

Search Strategy

Pubmed database

(Self burnin*) OR (Self immolation) OR (Burn*) OR (self mutilation) AND (self harm) OR (Intentio*) OR (Deliberate Self Harm) AND (suicide) OR (Attempt* Suicide) OR (Attempted Suicide) OR (parasuicide) AND (Pakistan)

Cochrane database

("Self burnin*") OR ("Self immolation") OR ("Burn*") OR ("self mutilation") AND ("self harm") OR ("Intentio*") OR ("Deliberate Self Harm") AND ("suicide*") OR ("Attempt* Suicide") OR ("parasuicide") AND (Pakistan)

For peer review only - http://bmjopen.bmj.com/site/about/guidelines.xhtml

Google Scholars

(“Self burnin*”) OR (“Self immolation”) OR (“Burn*”) OR (“self mutilation”) AND (“self harm”) OR (“Intentio*”) OR (“Deliberate Self Harm”) AND (“suicide*”) OR (“Attempt* Suicide”) OR (“parasuicide”) AND (Pakistan)

Pakmedinet

Suicide OR deliberate self-harm OR parasuicide OR self-harm OR attempted suicide AND burns OR self-mutilation OR Self-immolation AND Pakistan

Suicide | deliberate self-harm | parasuicide | self-harm | attempted suicide AND burns | self-mutilation | Self-immolation AND Pakistan

Grey literature for thesis and dissertation

OATD: Open Access Thesis and Dissertations.

For peer review only

For peer review only - http://bmjopen.bmj.com/site/about/guidelines.xhtml

Studies on Burns as a method of suicide and Self-harm in Pakistan

Author/year	Study Design	Year of Study	Source of data	Setting (City/province)

For peer review only

Suicide/SH cases	Total number of Burns cases	Number of Suicidal burns	Age

For peer review only

Gender	Marital status	Occupation	Place of burns	Extend of burns (TBSA)	Depth of burns

For peer review only

Reasons	Method/mechanism of burns	Outcome	Notes

For peer review only

BMJ Open

Suicide and Self-Harm by Burns in Pakistan: A Scoping Review Protocol

Journal:	*BMJ Open*
Manuscript ID	bmjopen-2023-080815.R2
Article Type:	Protocol
Date Submitted by the Author:	30-Jan-2024

Complete List of Authors:	Noorullah, Aisha; The Aga Khan University<br>Pirani, Shahina; The Aga Khan University<br>Bebbington, Emily; Bangor University, Centre for Mental Health and Society; Ysbyty Gwynedd, Emergency Department<br>Khan, Murad ; The Aga Khan University

**Primary Subject Heading**:	Mental health
Secondary Subject Heading:	Public health
Keywords:	Suicide & self-harm < PSYCHIATRY, PUBLIC HEALTH, PSYCHIATRY

SCHOLARONE™ Manuscripts

Suicide and Self-Harm by Burns in Pakistan: A Scoping Review Protocol

Principal Investigator

Dr. Aisha Noorullah
Senior Instructor
Department of Psychiatry
Aga Khan University, Stadium Road
PO Box 3500, Karachi-74800, Pakistan
Contact no. 9221-34930051
aisha.noorullah@aku.edu

Co. Investigators

Ms. Shahina Pirani
Research Specialist
Department of Psychiatry
Aga Khan University Pakistan
shahina.pirani@aku.edu

Dr. Emily Bebbington*
Welsh Clinical Academic Track (Public Health)
Specialist Registrar. Centre for Mental Health and Society,
Bangor University, Wrexham Academic Unit, Wrexham, LL13 7YP, UK.
e.bebbington@bangor.ac.uk

Dr. Murad M Khan
Professor Emeritus, Dept. of Psychiatry and Brain & Mind Institute
Aga Khan University Pakistan
murad.khan@aku.edu

***Corresponding authors**
Dr. Emily Bebbington, e.bebbington@bangor.ac.uk

For peer review only

ABSTRACT

Introduction

Suicide is a global public health problem. Self-inflicted burns are one of the most severe methods of suicide with high morbidity and mortality. Low and middle-income countries (LMICs) contribute 40% of all suicidal burns. Pakistan lacks comprehensive burns surveillance data which prevents the understanding of the magnitude of the problem. This scoping review aims to understand the scope of the problem of suicide and self-harm using burns in Pakistan and to identify knowledge gaps within the existing literature related to this specific phenomenon.

Methods and analysis

This scoping review will follow the methodological framework proposed by Arksey and O'Malley. We will search electronic databases (PubMed, Cochrane, Google Scholar, Pakmedinet), grey literature, and a reference list of relevant articles to identify studies for inclusion. We will look for studies on self-inflicted burns as a method of suicide and self-harm in Pakistan, published from the beginning till December 2023, in the English language. Two independent reviewers will screen all abstracts and full-text studies for inclusion. The data will be collected on a data extraction form developed through an iterative process by the research team and it will be analyzed using descriptive analysis.

Ethics and dissemination

Ethical exemption for this study has been obtained from the Institutional Review Board Committee of Aga Khan University Karachi Pakistan. The findings of the study will be disseminated by conducting workshops for stakeholders including Psychiatrists, Psychologists, counselors, general and public health physicians, and policymakers. Findings will be published in national and international peer-reviewed journals.

Keywords: suicide, self-harm, burns, scoping review, Pakistan

For peer review only - http://bmjopen.bmj.com/site/about/guidelines.xhtml

STRENGTHS AND LIMITATIONS OF THIS STUDY

- PRISMA protocol and scoping review reporting guidelines have been followed in the preparation of this manuscript and will be followed throughout the scoping review process.
- The review will be conducted in close collaboration between all authors. Any disagreement in the inclusion of studies will be discussed among the team until a consensus is reached. This process increases the validity and reliability of the review.
- Academic librarians will be collaborating to develop a search strategy and look for relevant papers in different databases.
- Only English language studies will be included. This is the dominant language used for the practice of medicine in Pakistan, but it may restrict the breadth of our results.

peer review only

For peer review only - http://bmjopen.bmj.com/site/about/guidelines.xhtml

BACKGROUND

Burns are a global public health problem, causing an estimated 180,000 deaths per year (1). Mortality from burn injuries is higher in low and middle-income countries (LMICs). Almost two-thirds of deaths occur in the WHO African and South-East Asia regions (1).

Self-inflicted burns are one of the most painful and violent methods of suicide, with high morbidity and mortality (2). Suicide by burning is a rare condition in high-income countries (0.06-1% of all suicides) but is more frequent in LMICs (accounting for as many as 40.3% of all suicides) (3). 77% of all suicides occurred in LMICs in 2019 (4).

Burn injuries can result in prolonged hospitalization and result in long-term physical and psychological sequelae (5). Non-fatal burns contribute to morbidity, such as disfigurement, and disability, leading to stigma and abandonment for the survivors (1). Burns are among the leading causes of disability-adjusted life-years (DALYs) lost in Asia (6).

Pakistan, a South Asian Islamic country, there are no official statistics on suicides at the national level (7). The WHO estimated there were 19,331 suicidal deaths and a rate of 8.9/100,000 people in 2019 (4). Until recently, suicides were treated as criminal acts in Pakistan, a religiously condemned and highly stigmatized. In December 2022, suicide and self-harms acts were decriminalized (8). However, it will take time for the abolition of the legal processes associated with them. Suicide and self-inflicted injuries are underreported due to religious, socio-cultural, and medicolegal processes. Suicidal burn injury is an important but under-researched area in Pakistan. Due to fear of societal stigma and legal issues, families withhold information about assault and self-harm and label it as accidental. This led to misclassification and undercounting of cases (9,10).

There are few systematic reviews and meta-analyses on suicidal burns from various parts of the world (11-14). These reviews revealed that highest prevalence of suicidal burns is found to be in South Asian countries including India, Iran, Iraq, Afghanistan, and Sri Lanka. In these studies, women represent many cases of suicidal burning with high mortality. Women who engage in suicidal self-burning were typically young, married, and mothers (2,11,12,14).

Existing literature reveals a systematic scoping review protocol on burns injury from South Asia (15). This review focused on terminology and methods used to differentiate the intent of hospital presenting burn injuries in South Asia. But there is no study that we are aware of that systematically investigates the demographics and outcomes of all suicidal burns in Pakistan.

For peer review only - http://bmjopen.bmj.com/site/about/guidelines.xhtml

The importance of reviewing the existing literature on burns studies in Pakistan lies in investigating the true extent of the burden imposed by suicidal burn injuries. It is crucial to explore the frequency, demographics, and outcomes reported to gain insights into this issue. Furthermore, the comparability of the findings in the literature remains undetermined, emphasizing the need for a scoping review to assess the availability and comparability of the existing research. Identifying specific groups that are prominently affected by these injuries may also highlight the necessity for further focused studies to address their special needs. The gravity of burns injuries, in terms of fatality (16), and the availability of the method with their profound impact on physical and mental health, differentiate these cases from other forms of self-harm, necessitates a comprehensive understanding of the under-researched subject matter in Pakistan. This scoping review will also serve as an initial step as part of the planning to commence a surveillance system in Pakistan by gauging the magnitude of the problem and its implications.

A search was conducted at Open Science Framework (OSF), Figshare, PubMed, and Prospero for systematic and scoping review on self-inflicted burn injuries in Pakistan, but no prior research specific to Pakistan was identified.

Aims & Objectives

This scoping review aims to understand the scope of the problem of suicide and self-harm using burns as a method of attempt across various age groups in Pakistan. It will also provide evidence to identify the knowledge gap in the existing literature on the phenomenon of interest. The specific objectives of the review include:

1. To assess the availability of data on the frequency of self-harm and suicide cases involving burns across various demographic factors in Pakistan.
2. To examine the methods commonly used for self-inflicted burns including self-harm and suicide in Pakistan.
3. To evaluate the outcomes of self-inflicted burns in Pakistan.

METHODS

We will conduct a scoping review to synthesize evidence on self-inflicted burns as a method of self-harm or suicide in Pakistan. This protocol has been written using Preferred Reporting Items for Systematic Reviews and Meta-Analyses extension for Scoping Reviews (PRISMA-ScR) (refer

For peer review only - http://bmjopen.bmj.com/site/about/guidelines.xhtml

supplementary file 1). This scoping review will follow the methodological framework proposed by Arksey and O'Malley (17) and amended by Levac et al. (18) This framework consists of six stages: (1) Identifying the Research Question, (2) Identifying Relevant Studies, (3) Study Selection, (4) Data Charting, (5) Data Analysis and Reporting the Results, (6) Consultation

The scoping review method is described considering the above six stages. This review has been registered with the Open Science Framework: https://doi.org/10.17605/OSF.IO/XA68S.

Stage 1: Identifying the Research Question

Based on the literature review, we formulated the following research questions:

Research question 1A

What is the frequency of self-harm and suicide cases involving burns in Pakistan?

Research question 1B

How is the frequency of self-harm and suicide cases involving burns distributed across different demographic factors such as age, sex, and marital status in Pakistan?

Research question 2A

What are common methods used for self-inflicted burns, including self-harm and suicide, in Pakistan?

Research question 2B

Are there any variations in the methods of self-inflicted burns based on demographic factors such as age, sex, and marital status in Pakistan?

Research question 3A

What are the short-term and long-term outcomes of self-inflicted burns in Pakistan?

Research question 3B

Are there any variations in the outcomes of self-inflicted burns based on demographic factors such as age, sex, and marital status in Pakistan?

Stage 2: Identifying Relevant Studies

For identifying the relevant studies, an academic librarian will assist in developing a robust search strategy. We will identify MESH keywords for each concept (burns, suicide, self-harm, intentional) included in the review question and use synonyms of key terms. Additionally, we will use Boolean operators with a combination of terms including (Self burning) OR (Self-immolation)

For peer review only - http://bmjopen.bmj.com/site/about/guidelines.xhtml

OR (Burns) OR (self-mutilation) AND (self-harm) OR (Intentional) OR (Deliberate Self-harm) AND (suicide) OR (Attempted Suicide) OR (parasuicide) AND (Pakistan) to look for relevant literature (refer supplementary file 2).

We will search in four databases including the National Library of Medicine's MEDLINE (PubMed), Google Scholar, Cochrane Library, and Pakmedinet. We will also search 'grey literature' that includes unpublished theses, conference papers, and other reports. In addition, we will employ an ancestry approach, checking the reference lists of retrieved articles for relevant references. We will search databases from the beginning until December 2023, published in the English language. The search strategies for each database will be similar in structure with similar search terms and synonyms. Additional keywords and sources identified during the process will be noted and reported. We will keep a separate record of the search results in a spreadsheet file including the date of search, search terms, and the number of retrieved articles. The search results will be exported into the reference management software Endnote and duplicate articles will be removed.

Eligibility Criteria

The selection of studies in the review will be based on thematic framework 'Population–Concept–Context (PCC)' recommended by Joanna Briggs Institute for scoping reviews (19), outlined under broader eligibility criteria. Consideration would be given to additional filters for type of publication and language preference (refer table 1)

Table 1: Inclusion and Exclusion Criteria for the Scoping Review

Themes	Inclusion criteria	Exclusion criteria
Population	Studies included populations with suicidal burns, in all genders, and across all ages.	Population with unintentional burns injuries.
Concept	- Studies focused on burns in general, where the intent was self-harm or suicide.<br><br>-Studies focused on self-harm or suicide where burns used as one of the methods.	Any article on burns that has no connection with suicide or self-harm.

For peer review only - http://bmjopen.bmj.com/site/about/guidelines.xhtml

	-Articles using different terminologies for suicidal burns (e.g. self-mutilation, self-immolation, self-inflicted burns)	
Context	-Studies conducted within the geographical boundaries of Pakistan.<br><br>-Studies conducted at hospital or community setting.<br><br>-Cases may be based on autopsy reports/forensic reports or Medico-legal reports.	Studies on Pakistanis residing outside the country.
**Additional Filters**		
Article Type	-Original research article with any study design quantitative or qualitative study.<br><br>-Case studies, Case series or Case report<br><br>- Editorial and Short communication<br><br>- Systematic, scoping, or rapid review.<br><br>-'grey literature' that includes unpublished theses and dissertations.	Media news
Reporting/ Language	-Articles published in the English language.	Other languages
Date of publication	Studies published from the beginning till December 2023, will be considered for this review.	Studies published after December 2023

Stage 3: Study Selection

Two researchers will conduct an exercise before initiating the search to ensure reliability in selecting articles for inclusion. This exercise will involve the independent screening of a random sample of 5% of the citations by the two independent reviewers. If the two researchers show low agreement, they will revisit the selection criteria.

The study selection process involves two steps. In the first step, two reviewers (SP and AN) will screen for the study titles and abstracts to determine eligibility for full-text screening

For peer review only - http://bmjopen.bmj.com/site/about/guidelines.xhtml

based on predefined inclusion criteria. A spreadsheet file will be used to record the search results, including the search date, terms used, and the number of retrieved articles. After exporting the search results into the Endnote reference management software, duplicate articles will be removed. In the second stage of the selection process, the full text of the articles will be retrieved and uploaded in an endnote. These will undergo independent screening by the same two reviewers (SP and AN) and any disagreements in eligibility between the reviewers will be resolved through discussion and involving a third researcher (MK).

We will use an adapted version of the PRISMA flow diagram to report the final numbers of included studies. At the full-text screening stage, we will record the reasons for excluding the studies.

Stage 4: Charting the Data

The data charting form has been developed in Microsoft Excel sheet after consultation with the subject expert and by reviewing the existing literature (refer supplementary file 3). This form included information on the source of the study, the place where the study was conducted, study design, time of the study, number of burns cases, number of suicidal burns, mechanism of burns, age, gender, marital status, reasons for burns, and outcome. We will pre-test the form and modify it accordingly.

Stage 5: Data Analysis and Reporting the Results

We will analyze the data descriptively using a data charting form. The study question included the frequency of suicidal burns across various demographics, methods commonly used for self-inflicted burns, and the outcome of injury will be the basis for analysis. We will present the characteristics of the study related to suicidal burns using tabulations or figures/flow chart where appropriate, to present a synthesis of key findings according to the scoping review objectives.

The researchers will report the scoping review according to the Preferred Reporting Items for Systematic Reviews and Meta-Analyses extension for Scoping Reviews (PRISMA-ScR) Checklist.

For peer review only - http://bmjopen.bmj.com/site/about/guidelines.xhtml

Stage 6: Consultations

In this final stage, we will consult with the subject experts (MK, EB) to validate the findings and add strength to the study. The subject expert will read the results and provide feedback, adding extra value to the scoping review.

Our scoping review work is in the study selection phase that began in November 2023. The authors anticipate that this review will be completed and submitted for publication by July 2024.

Patient and public involvement

There has been no patient or public involvement in the development of this protocol, nor will there be in the scoping review itself.

Coordination, Monitoring & Quality control

The study will ensure quality control by adhering to the standard protocols of conducting scoping reviews. This includes.

- Use of five stage framework by Arksey and O'Malley for conducting a literature review
- Development of a comprehensive search strategy
- Training and debriefing session for the two staff on conducting the scoping review.
- Independent review by two experienced team members.
- Frequent meetings with the research team to review the findings.

Ethical Considerations and Dissemination

Ethical exemption for this study has been obtained from the Institutional Review Board Committee of Aga Khan University Karachi Pakistan. We will disseminate the findings of the study by conducting workshops for stakeholders including Psychiatrists, Psychologists, counselors, general and public health physicians, and policymakers. Additionally, we will publish the findings in national and international peer-reviewed journals.

For peer review only - http://bmjopen.bmj.com/site/about/guidelines.xhtml

Authors' Contributions

AN & MK conceived the study and were involved in concept mapping and planning of the study. SP looked for a literature review and developed a study tool. SP & AN contributed to writing the study protocol. EB critically reviewed the protocol. MK intellectually contributed to the study. All authors have read and approved the final manuscript.

Funding

This work was supported by the South Asia Self-Harm Initiative. The South Asia Self-Harm initiative is funded by the Global Challenges Research Fund, UK Research and Innovation. Project number: MR/P028144/2. Principal Investigator: Prof. Catherine Robinson. The funder has not been involved in the development of this protocol.

Competing interests

None declared.

Patient consent for publication

Not required.

Ethics approval

Study protocol is exempted by the Aga Khan University Ethical Review Committee with ERC # 2023-9102-26269

For peer review only

For peer review only - http://bmjopen.bmj.com/site/about/guidelines.xhtml

REFERENCES

1. World Health Organization. Burns. Geneva: World Health Organization; 2023 https://www.who.int/news-room/fact-sheets/detail/burns

2. Canetto SS, Pouradeli S, Khan MM, Rezaeian M. Suicidal self-burning in women and men around the world: a cultural and gender analysis of patterns and explanations. InSuicide Risk Assessment and Prevention 2022 Sep 28 (pp. 1-28). Cham: Springer International Publishing.

3. Ahmadi A. Suicide by self-immolation: comprehensive overview, experiences and suggestions. Journal of burn care & research. 2007;28(1):30-41.

4. World Health Organization. Suicide worldwide in 2019: global health estimates. Geneva: World Health Organization; 2021.

5. Spronk I, Legemate C, Oen I, van Loey N, Polinder S, van Baar M. Health related quality of life in adults after burn injuries: a systematic review. PloS one. 2018; 13(5):e0197507.

6. Collier ZJ, McCool K, Magee III WP, Potokar T, Gillenwater J. 58 Burn injuries in Asia: a global burden of disease study. Journal of Burn Care & Research. 2022;43(Supplement_1):S40-1.

7. Asad N, Pirani S, Tariq S, et al. Patterns of suicide and self-harm in Pakistan: a retrospective descriptive study protocol. BMJ Open 2022;12:e064535. doi:10.1136/bmjopen-2022-064535

8. The Express Tribune. Law penalising suicide attempts abolished; December 23, 2022. https://tribune.com.pk/story/2392477/law-penalising-suicide-attempts-abolished

9. Naveed S, Tahir SM, Imran N, Rafiq B, Ayub M, Haider II, Khan MM. Sociodemographic characteristics and patterns of suicide in Pakistan: an analysis of current trends. Community mental health journal. 2023 Aug;59(6):1064-70.

10. Shahid M, Hyder AA. Deliberate self-harm and suicide: a review from Pakistan. International journal of injury control and safety promotion. 2008 Dec 1;15(4):233-41.

11. Laloë V. Patterns of deliberate self-burning in various parts of the world: a review. Burns. 2004;30:207–15.

For peer review only

For peer review only - http://bmjopen.bmj.com/site/about/guidelines.xhtml

12. Poeschla B, Combs H, Livingstone S, Romm S, Klein MB. Self-immolation: socioeconomic, cultural and psychiatric patterns. Burns. 2011;37:1049–57.

13. Hahn AP, Jochai D, Caufield-Noll CP, Hunt CA, Allen LE, Rios R, Cordts GA. Self-inflicted burns: a systematic review of the literature. J Burn Care Res. 2014;35:102–119.

14. Saadati M, Azami-Aghdash S, Heydari M, Derakhshani N, Rezapour R. Self-immolation in Iran: systematic review and meta-analysis. Bull Emerg Trauma. 2019;7:1–8.

15. Bebbington E, Ramesh P, Kakola M, McPhillips R, Bibi F, Hanif A, Morris N, Khan M, Poole R, Robinson C. Terminology and methods used to differentiate injury intent of hospital burn patients in South Asia: a systematic scoping review protocol. Systematic reviews. 2023;12(1):153.

16. Ali SA, Hamiz-Ul-Fawwad S, Al-Ibran E, Ahmed G, Saleem A, Mustafa D, Hussain M. Clinical and demographic features of burn injuries in karachi: a six-year experience at the burns centre, civil hospital, Karachi. Annals of burns and fire disasters. 2016;29(1):4.

17. Arksey H, O'Malley L. Scoping studies: towards a methodological framework. Int J Soc Res Methodol. 2005;8:19–3

18. Levac D, Colquhoun H, O'Brien KK. Scoping studies: advancing the methodology. Implement Sci. 2010;5:69

19. Peters MD, Marnie C, Tricco AC, Pollock D, Munn Z, Alexander L, McInerney P, Godfrey CM, Khalil H. Updated methodological guidance for the conduct of scoping reviews. JBI evidence synthesis. 2020;18(10):2119-26.

For peer review only

For peer review only - http://bmjopen.bmj.com/site/about/guidelines.xhtml

Preferred Reporting Items for Systematic reviews and Meta-Analyses extension for Scoping Reviews (PRISMA-ScR) Checklist

SECTION	ITEM	PRISMA-ScR CHECKLIST ITEM	REPORTED ON PAGE #
**TITLE**			
Title	1	Identify the report as a scoping review.	
**ABSTRACT**			
Structured summary	2	Provide a structured summary that includes (as applicable): background, objectives, eligibility criteria, sources of evidence, charting methods, results, and conclusions that relate to the review questions and objectives.	
**INTRODUCTION**			
Rationale	3	Describe the rationale for the review in the context of what is already known. Explain why the review questions/objectives lend themselves to a scoping review approach.	
Objectives	4	Provide an explicit statement of the questions and objectives being addressed with reference to their key elements (e.g., population or participants, concepts, and context) or other relevant key elements used to conceptualize the review questions and/or objectives.	
**METHODS**			
Protocol and registration	5	Indicate whether a review protocol exists; state if and where it can be accessed (e.g., a Web address); and if available, provide registration information, including the registration number.	
Eligibility criteria	6	Specify characteristics of the sources of evidence used as eligibility criteria (e.g., years considered, language, and publication status), and provide a rationale.	
Information sources*	7	Describe all information sources in the search (e.g., databases with dates of coverage and contact with authors to identify additional sources), as well as the date the most recent search was executed.	
Search	8	Present the full electronic search strategy for at least 1 database, including any limits used, such that it could be repeated.	
Selection of sources of evidence†	9	State the process for selecting sources of evidence (i.e., screening and eligibility) included in the scoping review.	
Data charting process‡	10	Describe the methods of charting data from the included sources of evidence (e.g., calibrated forms or forms that have been tested by the team before their use, and whether data charting was done independently or in duplicate) and any processes for obtaining and confirming data from investigators.	
Data items	11	List and define all variables for which data were sought and any assumptions and simplifications made.	
Critical appraisal of individual sources of evidence§	12	If done, provide a rationale for conducting a critical appraisal of included sources of evidence; describe the methods used and how this information was used in any data synthesis (if appropriate).	
Synthesis of results	13	Describe the methods of handling and summarizing the data that were charted.	

For peer review only - http://bmjopen.bmj.com/site/about/guidelines.xhtml

SECTION	ITEM	PRISMA-ScR CHECKLIST ITEM	REPORTED ON PAGE #
**RESULTS**			
Selection of sources of evidence	14	Give numbers of sources of evidence screened, assessed for eligibility, and included in the review, with reasons for exclusions at each stage, ideally using a flow diagram.	
Characteristics of sources of evidence	15	For each source of evidence, present characteristics for which data were charted and provide the citations.	
Critical appraisal within sources of evidence	16	If done, present data on critical appraisal of included sources of evidence (see item 12).	
Results of individual sources of evidence	17	For each included source of evidence, present the relevant data that were charted that relate to the review questions and objectives.	
Synthesis of results	18	Summarize and/or present the charting results as they relate to the review questions and objectives.	
**DISCUSSION**			
Summary of evidence	19	Summarize the main results (including an overview of concepts, themes, and types of evidence available), link to the review questions and objectives, and consider the relevance to key groups.	
Limitations	20	Discuss the limitations of the scoping review process.	
Conclusions	21	Provide a general interpretation of the results with respect to the review questions and objectives, as well as potential implications and/or next steps.	
**FUNDING**			
Funding	22	Describe sources of funding for the included sources of evidence, as well as sources of funding for the scoping review. Describe the role of the funders of the scoping review.	

JBI = Joanna Briggs Institute; PRISMA-ScR = Preferred Reporting Items for Systematic reviews and Meta-Analyses extension for Scoping Reviews.

* Where *sources of evidence* (see second footnote) are compiled from, such as bibliographic databases, social media platforms, and Web sites.

† A more inclusive/heterogeneous term used to account for the different types of evidence or data sources (e.g., quantitative and/or qualitative research, expert opinion, and policy documents) that may be eligible in a scoping review as opposed to only studies. This is not to be confused with *information sources* (see first footnote).

‡ The frameworks by Arksey and O'Malley (6) and Levac and colleagues (7) and the JBI guidance (4, 5) refer to the process of data extraction in a scoping review as data charting.

§ The process of systematically examining research evidence to assess its validity, results, and relevance before using it to inform a decision. This term is used for items 12 and 19 instead of "risk of bias" (which is more applicable to systematic reviews of interventions) to include and acknowledge the various sources of evidence that may be used in a scoping review (e.g., quantitative and/or qualitative research, expert opinion, and policy document).

From: Tricco AC, Lillie E, Zarin W, O'Brien KK, Colquhoun H, Levac D, et al. PRISMA Extension for Scoping Reviews (PRISMAScR): Checklist and Explanation. Ann Intern Med. 2018;169:467–473. doi: 10.7326/M18-0850.

For peer review only - http://bmjopen.bmj.com/site/about/guidelines.xhtml

Supplementary file 2 – SEARCH STRATEGY

MeSH keywords

Burns

Burns, Chemical
Burns, Electric
Burns, Inhalation

Suicide

Suicide, Attempted
Suicide, Completed

Suicide, Attempted

- Attempted Suicide
- Suicide Attempt
- Attempt, Suicide
- Parasuicide
- Parasuicides
- Self-Injurious Behavior

Suicide, Completed

- Completed Suicides
- Suicide, Fatal
- Completed Suicide
- Fatal Attempt
- Fatal Attempts
- Fatal Suicide
- Fatal Suicides
- Self-Injurious Behavior

Self-harm

- Behavior, Self-Injurious
- Self Injurious Behavior
- Self-Injurious Behaviors
- Intentional Self Injury
- Intentional Self Injuries

For peer review only - http://bmjopen.bmj.com/site/about/guidelines.xhtml

- Self Injury, Intentional
- Intentional Self Harm
- Self Harm, Intentional
- Deliberate Self-Harm
- Deliberate Self Harm
- Self-Harm, Deliberate
- Self-Injury
- Self Injury
- Self Harm
- Harm, Self
- Self-Destructive Behavior
- Behavior, Self-Destructive
- Self Destructive Behavior
- Self-Destructive Behaviors

Intentional

- Self-Injurious Behavior

Using Boolean operators:

(Self burning) OR (Self-immolation) OR (Burns) OR (self-mutilation) AND (self-harm) OR (Intentional) OR (Deliberate Self-harm) AND (suicide) OR (Attempted Suicide) OR (parasuicide) AND (Pakistan).

Search Strategy

Pubmed database

(Self burnin*) OR (Self immolation) OR (Burn*) OR (self mutilation) AND (self harm) OR (Intentio*) OR (Deliberate Self Harm) AND (suicide) OR (Attempt* Suicide) OR (Attempted Suicide) OR (parasuicide) AND (Pakistan)

Cochrane database

("Self burnin*") OR ("Self immolation") OR ("Burn*") OR ("self mutilation") AND ("self harm") OR ("Intentio*") OR ("Deliberate Self Harm") AND ("suicide*") OR ("Attempt* Suicide") OR ("parasuicide") AND (Pakistan)

For peer review only - http://bmjopen.bmj.com/site/about/guidelines.xhtml

Google Scholars

(“Self burnin*”) OR (“Self immolation”) OR (“Burn*”) OR (“self mutilation”) AND (“self harm”) OR (“Intentio*”) OR (“Deliberate Self Harm”) AND (“suicide*”) OR (“Attempt* Suicide”) OR (“parasuicide”) AND (Pakistan)

Pakmedinet

Suicide OR deliberate self-harm OR parasuicide OR self-harm OR attempted suicide AND burns OR self-mutilation OR Self-immolation AND Pakistan

Suicide | deliberate self-harm | parasuicide | self-harm | attempted suicide AND burns | self-mutilation | Self-immolation AND Pakistan

Grey literature for thesis and dissertation

OATD: Open Access Thesis and Dissertations.

Suicide, deliberate self-harm, self-harm, attempted suicide, burns, self-burning, Pakistan

For peer review only

For peer review only - http://bmjopen.bmj.com/site/about/guidelines.xhtml

Studies on Burns as a method of suicide and Self-harm in Pakistan

Author/year	Study Design	Year of Study	Source of data	Setting (City/province)

For peer review only

Suicide/SH cases	Total number of Burns cases	Number of Suicidal burns	Age

For peer review only

Gender	Marital status	Occupation	Place of burns	Extend of burns (TBSA)	Depth of burns

For peer review only

Reasons	Method/mechanism of burns	Outcome	Notes

For peer review only
